# Pulse Signal Analysis Based on Deep Learning Network

**DOI:** 10.1155/2022/6256126

**Published:** 2022-09-15

**Authors:** Quanyu E

**Affiliations:** Department of Electrical and Electronic Engineering, The University of Hong Kong, Hong Kong, 999077 Hong Kong, China

## Abstract

Pulse signal is one of the most important physiological features of human body, which is caused by the cyclical contraction and diastole. It has great research value and broad application prospect in the detection of physiological parameters, the development of medical equipment, and the study of cardiovascular diseases and pulse diagnosis objective. In recent years, with the development of the sensor, measuring and saving of pulse signal has become very convenient. Now the pulse signal feature analysis is a hotspot and difficulty in the signal processing field. Therefore, to realize pulse signal automatic analysis and recognition is vital significance in the aspects of the noninvasive diagnosis and remote monitoring, etc. In this article, we combined the pulse signal feature extraction in time and frequency domain and convolution neural network to analyze the pulse signal. Firstly, a theory of wavelet transform and the ensemble empirical mode decomposition (EEMD) which is gradually developed in recent years have been used to remove the noises in the pulse signal. Moreover, a method of feature point detection based on differential threshold method is proposed which realized the accurate positioning and extraction time-domain values. Finally, a deep learning method based on one-dimensional CNN has been utilized to make the classification of multiple pulse signals in the article. In conclusion, a deep learning method is proposed for the pulse signal classification combined with the feature extraction in time and frequency domain in this article.

## 1. Introduction

**Figure 1:**
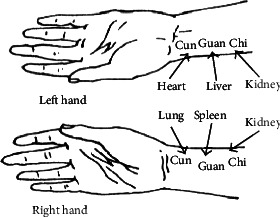
Traditional Chinese medicine pulse diagnosis diagram.

In the past two thousand years, pulse diagnosis, as one of the most important methods of disease diagnosis, has taken a significant part in traditional Chinese medicine. Traditional Chinese medicine believes that changes in the state of internal organs such as the internal organs of the human body or the limbs and facial features will produce a fixed pulse. By touching the pulse of the patient, the internal changes of various organs of the body can be judged, thereby diagnosing the physical condition and the location of the disease. Studies have shown that the pulse signal mainly comes from the heart. Under the periodic pulsation of the heart and the elastic expansion and contraction of the vascular system, blood will flow through various organs of the body [1], and eventually, this blood will be collected on the human wrist. Fingertips, ear tips, and other parts form a pulse signal; therefore, the human pulse signal contains not only heart health disease information but also blood viscosity, blood vessel wall elasticity, blood flow velocity, and other physiological and pathological information related to other human organs [2, 3]. Therefore, whether based on the traditional Chinese medicine perspective or the modern scientific perspective, the study of pulse signals has great theoretical value and practical significance.  Figure 1 shows some typical pulse waves in traditional Chinese medicine.

**Figure 2:**
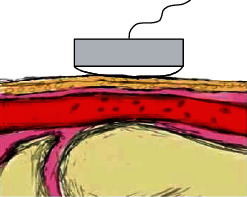
Mechanism of pressure sensor.

By studying the mechanism of pulse signal generation, pressure pulses and volume pulse signals reflecting the blood pressure or volume information of human blood vessels can be successfully extracted. Compared with the volume pulse signal detection process, the pressure pulse signal detection has many advantages such as easy operation, reliable performance, safety, noninvasiveness, and strong adaptability. Therefore, it has been widely concerned by domestic and foreign researchers and engineers [4, 5]. As shown in Figure 2, the pressure sensor is placed at the superficial artery (such as the radial artery) to detect changes in the blood vessel pressure at the artery to obtain the pulse wave signal. Since the pulsation change curve of blood vessel diameter with time is approximately similar to the pulsation change curve of intravascular pressure, the curve of the change of blood vessel diameter with time detected by various pressure sensors clinically reflects the pulsation change of intravascular pressure, thus achieving noninvasive. The pressure sensors that are widely used in piezoelectric, piezoresistive, piezomagnetic (inductive), etc. [6, 7]. The pressure sensor has become the most widely used sensor in the current pulse wave signal acquisition application due to its advantages such as easy manufacture, high power-electricity conversion efficiency, high accuracy, and similar use form to clinical pulse diagnosis [8].

The pulse signal is a nonlinear, nonstationary signal with an approximate period. Initially, it was mainly time-domain analysis, such as analysis through intuitive parameters of the shape, peak value, angle, and area of ascending and descending branches of the pulse waveform, but a lot of practice shows that the information contained in the pulse signal is multifaceted. It is difficult to find the rich information that has involved in the pulse signal by the intuitive analysis method alone, thus promoting the development of the pulse signal analysis method in a more comprehensive and diversified direction. In recent years, the methods that have been commonly used in pulse signal analysis are time domain analysis and time-frequency joint analysis method.

**Figure 3:**
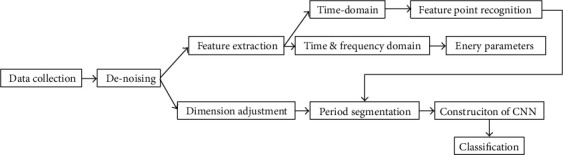
A framework for pulse signal analysis.

Figure 3 shows the structure diagram of the article. It summarizes the pulse signal analysis in four aspects. This dissertation starts from the collected 10 types of pulse signals and is aimed at analyzing the pulse signal through the EEMD, stationary wavelet transform, and HHT signal denoising methods, deeply excavate a large number of parameters. After the pulse signal analysis process, the preprocessed signal can be gained. Finally, through the CNN, the preprocessed pulse signal can be processed to realize the classification and recognition of various pulse signals.

The purpose of this study is to classify the collected pulse signals through a self-made pulse sensor and to cooperate with traditional Chinese medicine to extract the corresponding characteristics of the pulse through the pulse and to predict and analyze some basic diseases. The novelty of this research is that a set of standard database with 30 kinds of human pulses is established by using the touch training machine of the Faculty of Chinese Medicine of the University of Hong Kong, which can be used for classification and disease prediction.

## 2. Preprocessing Methods

### 2.1. Related Work

In 2021, the article in [9] mentioned a method about a multiscale CNN-CRF framework for environmental microorganism image segmentation. This method requires 420 scenes of EM images; however, it is difficult to gain a huge number of EM images. In order to increase the number of the dataset, 105 training images with the revolution of 256 × 256 pixels have been rotated by 0, 90, 180, and 270 degrees and mirroring and then divided into patches with 8 × 8 pixels. After this procedure, the 107520 patches have been obtained.

The paper [10] comes up with a state-of-the-art review for gastric histopathology image analysis approaches and future development. This work pointed out that machine learning needs a large number of datasets. The augmentation approaches include rotation, reverse, and scale transform, such as the degree rotation, colour vibrance, adding noise, and the reverse transformation.

### 2.2. Pulse Signal Data Preprocessing

The collected pulse signal usually contains all kinds of noise interference, which will cause different degrees of signal distortion and affect the quality of pulse signal under test. Therefore, before performing pulse signal analysis and processing, it needs to be preprocessed to obtain a high-quality pulse signal.

The noise interference that affects the quality of pulse signal acquisition mainly includes baseline drift and high-frequency noise, which can usually be filtered by software. The respiration of the human body and the movement of the hands on the period of signal acquisition will lead the baseline drift of the pulse signal, causing the lowest point of the pulse waveform of each cycle to deviate from the same horizontal line. It is a low-frequency signal that changes slowly and is easily mixed in the useful pulse signal [11, 12]; high-frequency noise mainly comes from random noise and environmental interference, such as electromagnetic interference of electronic equipment and thermal noise of electronic devices. These high-frequency or low-frequency noises will affect the accuracy of pulse signal measurement, so it is of great significance to take the necessary measures to filter out noise interference.

#### 2.2.1. Denoising Based on Wavelet Transform

**Figure 4:**
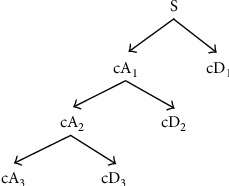
Three-layer wavelet decomposition tree.

Wavelet transform is a time-frequency localized analysis method based on multiresolution theory. It can study any details of the signal and is an important method for signal time-frequency analysis. The specific decomposition process can be seen in Figure 4. The number of decomposition layers is determined by the analyzed data and the needs of the user [13].

The initial signal is decomposed into *N* layers, and the signal can be expressed as the superposition of the high frequency part and the low frequency part, which can be expressed by the following formula:

(1) $$\begin{array}{c} S={cA}_{1}+{cD}_{1}={cA}_{2}+{cD}_{2}+{cD}_{1}=\cdots ={cA}_{n}+{cD}_{n-1}+\cdots +{cD}_{1}. \end{array}$$

Among them, the low-frequency coefficient and the high-frequency coefficient indicate that the frequency range of the signal is different, the wavelet details representing high-frequency noise are mainly distributed in the high-frequency range, and the wavelet details representing baseline drift are mainly distributed in the low-frequency range. By filtering out the wavelet details in the frequency range where the high-frequency noise and baseline drift are located, the filtered pulse signal can be obtained by wavelet reconstruction.

The wavelet details between the maximum decomposition level of a single cycle of a periodic signal and the maximum decomposition level of the entire multicycle signal represent the baseline of the periodic signal [14]. Use db7 as the wavelet basis function and the maximum resolution scale that can be selected for wavelet decomposition of pulse signal is

(2) $$\begin{array}{c} N=\text{floor}\left|\log_{2}\left(L\right)\right|=\text{floor}\left|\log_{2}\left(1000\right)\right|=9, \end{array}$$

where *L* stands for the length of the pulse wave signal and floor represents the rounding down operation.

To calculate the maximum number of decomposition layers of a pulse signal in a single period, it is vitally important to calculate the number *M* of pulse waves in a piece of pulse data, which can be obtained by the differential threshold method or the Fourier transform method. The maximum decomposition level of a single-cycle signal is

(3) $$\begin{array}{c} N_{1}=\text{Ceil}\left|\log_{2}\left(\frac{L}{M}\right)\right|=\text{Ceil}\left|\log_{2}\left(\frac{1000}{7}\right)\right|=8. \end{array}$$

Among them, ceil means rounding up. After calculating *N* and *N*1, the number of decomposition layers where the baseline drift is located can be determined. At the same time, the pulse signal is mainly distributed within 20 Hz. Therefore, by filtering out high-frequency noise greater than 20 Hz and low-frequency baseline drift noise, you can get a more ideal pulse signal.

**Figure 5:**
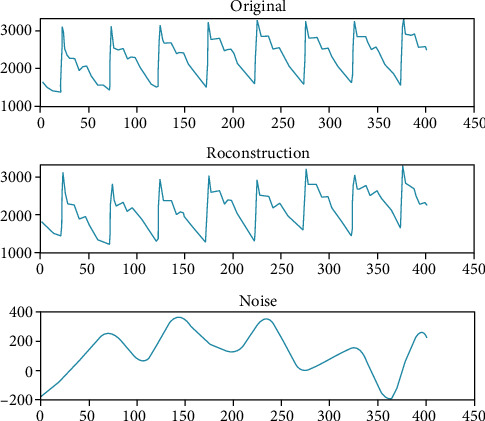
Denoising result based on wavelet transform.

From Table 1, we can know that the noise greater than 20 Hz is mainly distributed in the wavelet details of layer 1 and layer 2. Setting the high-frequency wavelet coefficients and low-frequency wavelet details to zero, that is *cD*_1_, *cD*_2_, *cD*_8_, *cD*_9_ and *cA*_9_ in Table 1. Then, reconstruct the signal to gain the denoised signal. The original signal, reconstructed signal, and error curve are shown in Figure 5.

#### 2.2.2. Denoising Using HHT Algorithm Based on EEMD

Hilbert-Huang transform (HHT) is another important time-frequency analysis way, which mainly includes two aspects, EEMD decomposition and Hilbert spectrum analysis. By performing the Hilbert transform on the IMF component gained by EMD decomposition, the instantaneous frequency and instantaneous amplitude of the signal will be gained [15].

The decomposition process of EEMD method [16]: add the mean value to *M* times (*M* > 1) in the initial signal *x*(*t*), and the standard deviation of the amplitude is constant Gaussian white noise *n*_*i*_(*t*)(*i* = 1 ~ *M*), namely,

(4) $$\begin{array}{c} x_{i}\left(t\right)=x\left(t\right)+n_{i\left(t\right)}. \end{array}$$

EMD decomposition is performed on *x*_*i*_(*t*) to obtain K IMF components denoted as *c*_*i*_(*t*)(*j* = 1 ~ *KK*) and remainder *f*_*i*_(*t*), where *c*_*i*_(*t*) represents the *j*-th IMF component gained when the Gaussian white noise is added for the *i* times. Utilizing the theory that the statistical mean value of the uncorrelated random sequence is zero, the IMF component *c*_*i*_(*t*) which related to the above step is averaged to offset the influence of adding Gaussian white noise in the real IMF multiple times. The IMF is

(5) $$\begin{array}{c} c_{j}\left(t\right)=\frac{1}{M}\overset{N}{\underset{i=1}{\sum }}c_{ij}\left(t\right), \end{array}$$

where *c*_*i*_(*t*) stands for the *j*-th IMF gained by EEMD decomposition of the initial signal [17] and *M* refers to the number of added white noise sequences.

Hilbert transform of the time domain signal*x*(*t*)is defined as

(6) $$\begin{array}{c} \hat{x}\left(t\right)=H\left|x\left(t\right)\right|=\frac{1}{\pi }\overset{+\infty }{\underset{-\infty }{\sum }}x\left(\tau \right)\frac{1}{t-\tau }d\tau . \end{array}$$

As we all know from the formula above that the Hilbert transform of *x*(*t*) can also be expressed as a convolution form of *x*(*t*) and (*πτ*)^−1^, namely,

(7) $$\begin{array}{c} \hat{x}\left(t\right)=\frac{1}{\pi \tau }\times x\left(t\right). \end{array}$$

Fourier transform of the two sides of the formula is as follows:

(8) $$\begin{array}{c} F\left|\hat{x}\left(t\right)\right|=\frac{1}{\pi }F\left|\frac{1}{\tau }\right|f\left|x\left(t\right)\right|=jsgh\left(f\right)\cdot F\left|x\left(x\right)\right|. \end{array}$$

The Hilbert transform of the signal *x*(*t*) will be gained by inverse Fourier transform of the formula.

Hilbert transform of the IMF component is as follows:

(9) $$\begin{array}{c} \hat{c_{i}}\left(t\right)=H\left|c_{i}\left(t\right)\right|=\frac{1}{\pi }\overset{+\infty }{\underset{-\infty }{\sum }}c_{i}\left(\tau \right)\frac{1}{t-\tau }d\tau . \end{array}$$

According to the characteristics of the Hilbert transform, *c*_*i*_(*t*) can be regarded as the real part, and *c*_*ı*_(*t*) can be regarded as the imaginary part, and the two parts can combine as the analytical signal *z*_*i*_(*t*):

(10) $$\begin{array}{c} z_{i}\left(t\right)=c_{i}\left(t\right)+j\hat{c_{i}}\left(t\right)=a\left(t\right)e^{j\theta \left(i\right)}. \end{array}$$

**Figure 6:**
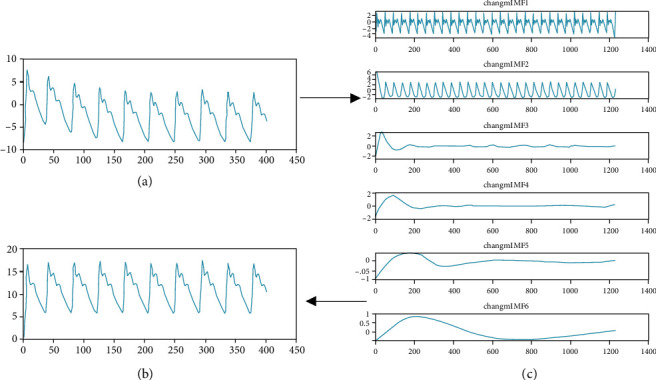
The debaseline drift of pulse signal: (a) original pulse wave; (b) denoised pulse wave; (c) each IMF component.

Filter the IMF component which instantaneous frequency < 0.5 Hz and >20 Hz, and combine the other IMF component to get the denoising signal. The whole debaseline drift process is shown in Figure 6.

## 3. Feature Extraction Methods of Pulse Signal

**Figure 7:**
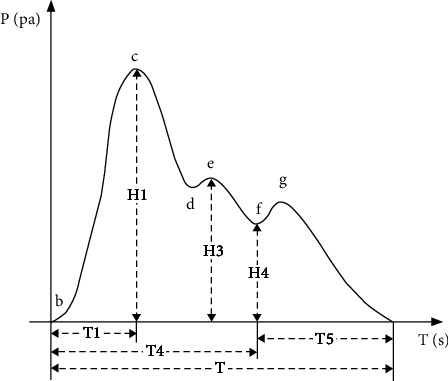
Typical pulse waveform.

Due to the superficial position of the radial artery, the detection is the most convenient. It is the same as the clinical pulse diagnosis position of traditional Chinese medicine, and the radial artery is closest to the peripheral blood vessel. At present, the pulse wave is mainly measured from the radial artery. According to the mechanism of pulse wave formation, it can be divided into different heartbeat stages. The complete pulse wave waveform under normal physiological conditions is shown in Figure 7.

A complete pulse wave waveform consists of ascending branches and descending branches [18], which mainly includes 5 feature points. The meaning of each feature point is as follows.

Point b, the opening point of the aorta, which is the starting point of a pulse wave period, is the valley point of the pulse wave pattern, which marks the left ventricle contraction. Point c is the main wave, which is the maximum point in the pulse wave waveform. Point e, the tidal wave, also known as the front wave of the weighting wave, is located on the descending branch of the waveform, generally after the main wave. Point f, the through valley, is the dividing point between systole and diastole, located before the dicrotic wave and after the tidal wave. Point g is the striking wave crest, and it is a protruding wavelet, located after point f.

### 3.1. Pulse Wave Feature Point Recognition

For the sake of recognizing a total of three feature points from the aortic opening point (point b), the main wave peak (point b), the tidal wave (point e), and the trough valley (point f) in the pulse wave waveform, this paper uses a pulse wave feature point automatic recognition algorithm.

The characteristics of the pulse waveform are mainly represented by the extreme points and inflection points of the waveform. Therefore, the use of the differential threshold method can easily extract the pulse characteristic parameters.

The first-order and second-order differential processing of the pulse signal can obtain the maximum point of the signal. Since the height of the main wave of the pulse signal is the maximum point in a cycle, set the maximum value of the signal greater than *m* times by adjusting the value of parameter *m* which can get better recognition effect. In the same way, when the height of the dicrotic wave is set to be greater than *n* times and less than *m* times the maximum value, a better recognition effect can also be obtained.

**Figure 8:**
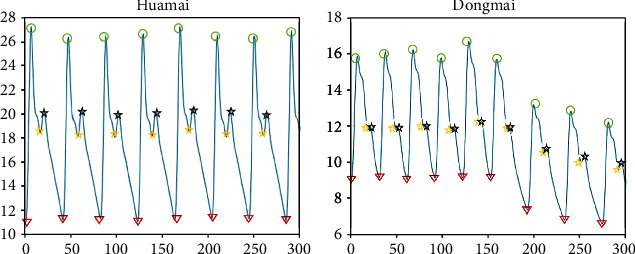
Result diagram for discovering feature points based on differential threshold method.

Through the transformation law of the first-order and second-order differential signals, the minimum point of the signal is found. When the maximum figure of the signal with the height of the lowering gorge is greater than *k* times, a better feature parameter extraction effect can be obtained. At the same time, by observing the collected pulse signal, we can know that the pulse signal is distributed around the minimum point of the starting point. When the differential threshold method is used to extract the position information of the point, it is easy to introduce confusion and cannot accurately distinguish the pulse signal feature points. When the first-order differential value at this point is required to be greater than a certain value *a*, better results can be obtained. The result diagram for discovering feature points based on differential threshold method is shown in Figure 8.

### 3.2. Characteristic Parameters in Time and Frequency Domain

Through wavelet transform, not only the denoising of the pulse signal can be achieved but also the characteristic parameters of the waveform in the time or frequency domain can be extracted. As a result, it has been commonly concerned by researchers. Shi et al. pointed out a signal feature detection way through wavelet transform [19], and simulation proved that the method has a higher detection accuracy and a more accurate positioning effect. Hongbiao proposed a feature extraction method suitable for nonstationary pulse signals [20]. Use wavelet decomposition to realize the classification and identification of healthy people and patients with atherosclerosis.

Perform 5-layer decomposition on samples 1, 2, and 3 to obtain decomposition coefficients *cA*_5_*cD*_5_, *cD*_4_ , *cD*_3_, *cD*_2_, and *cD*_1_. According to the law of wavelet decomposition, the frequency ranges corresponding to *cA*_5_*cD*_5_, *cD*_4_, *cD*_3_, *cD*_2_, and *cD*_1_ are 0-3.13, 3.13-6.25, 6.25-12.5, 12.5-25, 25-50, and 50-100 Hz. Since information greater than 20 Hz is filtered out during preprocessing, the amplitudes of *cD*_2_ and *cD*_1_ are close to zero. At the same time, we can know from the wavelet decomposition diagram that the amplitude of the wavelet coefficients of low-frequency information is large, and the amplitude of the wavelet coefficients of high-frequency information is small.

Then, according to the relationship between the wavelet coefficients and the signal energy distribution, the square sum of the wavelet coefficients of each layer is calculated. We can get the feature vector *T*:

(11) $$\begin{array}{c} T=\left[E_{cA5},E_{cD5},E_{cD4},E_{cD3},E_{cD2},E_{cD1}\right]. \end{array}$$

Furthermore, the normalized feature vector *TT*_0_ can be obtained:

(12) $$\begin{array}{c} T_{0}=\frac{\left[E_{cA5},E_{cD5},E_{cD4},E_{cD3},E_{cD2},E_{cD1}\right]}{E_{0}}. \end{array}$$

We can see from Table 2 that the energy ratios of data 1-6 at 0-6.25 Hz are all higher than 99%, indicating that the collected pulse signals are all from healthy people. At the same time, using statistical methods to analyze the wavelet coefficients in each layer in detail, we can study their relationship with specific diseases and promote the objective development of pulse diagnosis.

## 4. Experiments and Results

### 4.1. Experiment Setting

#### 4.1.1. Pulse Signal Dataset

The dataset consists of the raw pulse data collected by the homemade pulse sensing device of Lee Ultrasound Lab from the palpation training machine in Traditional Chinese Medicine School of The University of Hong Kong. There are 10 types of pulse waves (or Mais) which contain in the dataset. The names of those Mais are as follows: Changmai, Chimai, Dongmai, Huamai, Huanmai, Jimai, Pingmai, Shimai, Weimai, and Xuanmai. Every type of Mais was collected in 3 different applied pressures. There is no quantitative standard for the applied pressure of the palpation training machine, nor the quantitative math model for the applied pressure and the pulse signal. Therefore, the 3 applied pressures were not unified cross different Mais. When no extra pressure forced on the sensor, there is still an initial pressure of the sensor. The initial pressure was recalled as the baseline. It was caused by the produce process that needs to be subtracted from the collected signal. Each data signal contains two types of variables. The *x* axis (the horizontal axis) represents the pressure sample points which units is “Pa.” The *y* axis (the vertical axis) represents the time points when collecting the corresponding pressure points.

#### 4.1.2. Experimental Environment

The preprocessing part of the experiment in this paper is implemented on MATLAB R2019a, and the deep learning part is implemented with Python 3.7. The deep learning uses CNN network. The CNN model is built and trained on TensorFlow on an 8 GB memory computer.

The experiment runs on macOS 10.15.

### 4.2. Training and Test Data Setting

Preprocess the collected signals in the above process to denoise and solve the problem of baseline drift. Here, the wavelet transform method which has been mentioned in Chapter 2.2.1 is used for preprocessing.

#### 4.2.1. Pulse Data Dimension Adjustment

During signal acquisition, the saved data variables are time and pressure, because the hardware reading is not absolutely uniform, resulting in different time intervals. When performing deep learning on data, it is necessary to ensure that each input data has the same length, so cubic spline interpolation is performed on the original data to expand the original data volume tenfold. After that, resampled the interpolated data; that is, 50 time slices (50 points per second) are intercepted every second, thereby ensuring that the time interval of the sampling points is uniform.

#### 4.2.2. Pulse Segmentation and Augmentation

Because the collected signals have different lengths, the signals must be segmented. Use the feature points of the pulse signal which has been mentioned in Chapter 2.2.1 to find the lowest point of the pulse signal (that is, point b, the opening point of the aorta), use it as the starting point of the signal, and cut the resampled data. Intercept the pulse signal with a length of 6 seconds (that is, 300 time slices). At the same time, due to insufficient data volume and in order to not waste data, prevent the overfitting phenomenon, and better describe each type of pulse type, we cross-intercept the pulse signal by intercepting a segmentation of pulse signal in every other feature point (point b) to expand the dataset.

**Figure 9:**
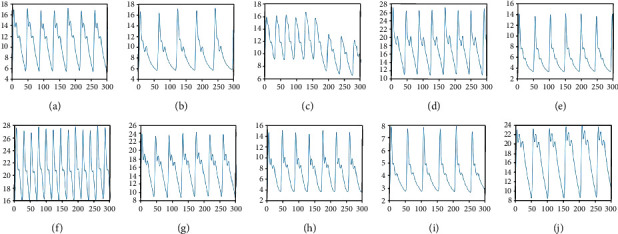
Database of the pulse wave: (a) Changmai; (b) Chimai; (c) Dongmai; (d) Huamai; (e) Huanmai; (f) Jimai; (g) Pingmai; (h) Shimai; (i) Weimai; (j) Xuanmai.

Through the operations above, 677 sets of pulse data have been obtained in the experiment: Changmai (59), Chimai (49), Dongmai (79), Huamai (63), Huanmai (63), Jimai (130), Pingmai (54), Shimai (60), Weimai (50), and Xuanmai (70). As we can see from Figure 9, it is the database of the pulse signal for each pulse wave.

#### 4.2.3. Arrangement of Datasets

The collected 10 types of pulse signals have been divided into 677 sets of data. And then, the dataset is randomly allocated into training set and test set by the ratio of 7 : 3. As a result, the training set contains 473 sets of data, and the test set involves 204 sets of data. The arrangement of the training and test datasets can be seen in Table 3.

### 4.3. Related Work

**Figure 10:**
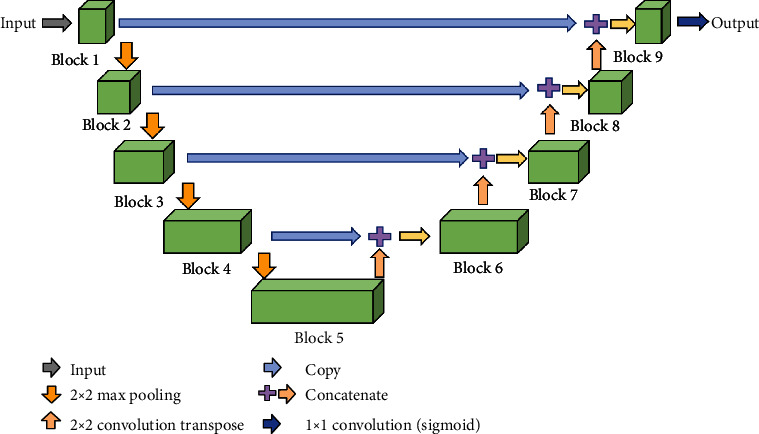
The architecture of mU-Net.

In [19], the authors mentioned a multiscale CNN-CRF framework for environmental. In this work, a novel method called mU-Net-BXs is utilized to optimize the adaptability of U-Net convolution neural network. The original inception using different size convolution filters of 1 × 1, 3 × 3, and 5 × 5 (called BOLCK-I) is the direct way to optimize the adaptability of U-Net. Inspired by Inception V2 and Inception V3, a 5 × 5 convolution filter can be replaced by a sequence of two 3 × 3 convolution filter, and a 7 × 7 convolution filter actually resembles a sequence of three 3 × 3 convolution filters (called BLOCK-II). In the same way, a 3 × 3 convolution filter can also be replaced by a sequence of 1 × 3 and 3 × 1 convolution filters as BLOCK-III. And then, deploy the BLOCK-III in mU-Net architecture. The whole architecture of the network U-Net convolution neural network is shown in Figure 10.

In [21], the authors proposed an approach of the identification of COVID-19 samples from X-ray images using deep learning in which collected COVID CXR images from the GitHub repository with various sizes of pixels and kept target size to the same pixel. Then, apply VGG series, Xception, ResNet V1 and ResNet V2 series, Inception series, DenseNet series, and MobileNet networks in the transfer learning process, where the weights are pretrained on ImageNet dataset.

### 4.4. Construction of the Classification of the Pulse Signal Algorithm Based on the CNN Model

The constructed convolutional neural network consists of two parts, the convolutional layer and the fully connected layer. The convolutional layer includes four convolutional layers and two pooling layers. In the connection layer, the selected activation function is the softmax function. The loss function selects the categorical_crossentropy function suitable for multiclassification, and the metric selects the commonly used accuracy.

**Figure 11:**
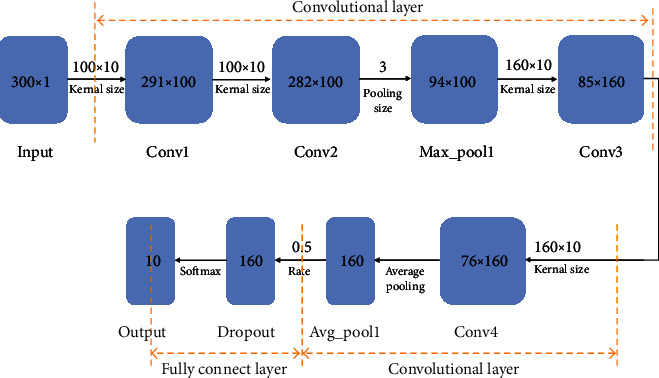
CNN model framework based on pulse classification.

The whole model framework is shown in Figure 11; it contains 2 parts. For input data, the data has been preprocessed, and each data record contains 300 time slices (the pulse wave data has been divided into 6 seconds pulse wave; each second includes 50 time slices). In each time slices, three values for the left horizontal axis, right horizontal axis, and vertical axis will be saved. This will produce a 300 × 1 matrix. The data should be put into the model with a size of 300. The first layer of the network should be embedding the data to its initial size, that is, 300 × 1.

The first convolutional layer: the first layer can be seen as a filter (or feature detector) with a size of 10 (also named the kernel size). However, only one filter is not enough for the model to learn all the features in the first convolution layer, so we select 100 feature detectors. This makes us to train 100 different functions at the first layer of the model. The result of this CNN layer is a 291 × 100 matrix. Each column of the output matrix contains a filter weight. Using the defined kernel size and considering the length of the input matrix, each feature detector involves 291 weights.

Second convolutional layer: results from the last layer should put into the second convolution layer. We can select 100 different feature vectors to train at this layer as well. Having the same steps as the last layer, the size of the result will be 282 × 100.

Maximum pool layer: for the sake of reducing the intricacy of the result and avoiding the data of overfitting, the pool layer is usually used after the CNN layer. In this study, we select 3 sizes. As a result, the input matrix's size is 3 times larger than the output matrix.

Third and fourth convolution layers: for the sake of the model to have higher level of functions, the next is another sequence of 2 convolution layers. However, the length of the feature detector is still 10; we select 160 feature detectors. As a result, the output matrix after these two layers is a 76 × 160 matrix.

Average pooling layer: the reason for this layer is make one more layer of pooling to further avoid overfitting. Meanwhile, instead of taking the max value, the average of the two weights in the CNN model is taken. The size of the output matrix is 1 × 160 neuron matrix. Each feature detector has only one weight left in this layer of neural network.

Dropout layer: the dropout layer will randomly assign 0 weights to the neurons in the network. When the ratio we select is 0.5, the weight of 50% of neurons is zero. After this step, the CNN model will become less sensitive to small changes of data. Therefore, it should further improve the accuracy of our invisible data. The result of this layer is still a 1 × 160 neuron matrix.

Fully connected layer: the model has to predict 10 categories (“Changmai,” “Chimai,” “Dongmai,” “Huamai,” “Huanmai,” “Jimai,” “Pingmai,” “Shimai,” “Weimai,” “Xuanmai”). The activation function we select is softmax. It makes the sum of the total 10 results of the CNN model to be 1. Therefore, the result will stand for the probability of each of the ten types.

### 4.5. Analysis of the Experimental Results

**Figure 12:**
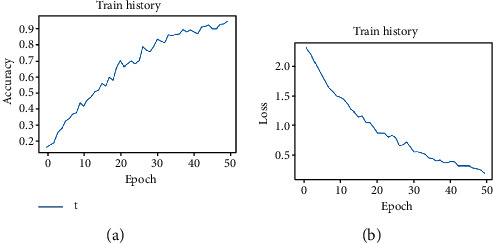
Result of the test set of the training model: (a) accuracy of the model in 50 times; (b) loss rate of the model in 50 times.

Input the processed data into the built CNN model for training and testing. The training times for the training set are set to 50 times (epoch = 50).  Figure 12 shows the accuracy and loss rate of the training set during the training process. As the information shown in Figure 12, when the training time is more than 40 times, the accuracy rate gradually stabilizes at around 90%. The final accuracy rate stabilizes at around 94%, and the loss rate decreases to within 0.2 with the increase of the training times.

Then, put the test set data into the trained CNN model, and the accuracy rate of the test set is 94.12%. For the training result, we select precision, recall, and F1 score those three evaluation parameters to evaluate the classifier performance. The equation of the three parameters is shown in Table 4.

**Figure 13:**
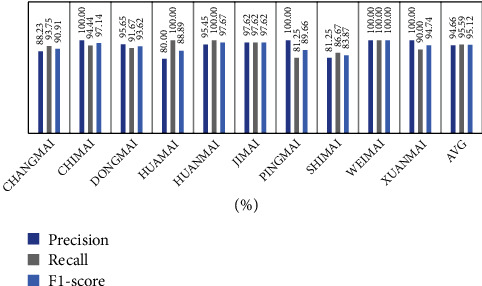
Classification result of different pulse wave based on the CNN model.

Figure 13 shows that the evaluation parameters of the test set. From Figure 13, we know that the accuracy value, recall rate, and F1 score of each group are all above 80%, the individual pulse wave accuracy rate can reach 100%, and the average recall rate and F1 score are above 95%; the average precision rate is above 94%. The average of the three evaluation parameters is not much different. In conclusion, the model has good performance in accuracy and stability.

The test set contains a total of 204 sets of data, which are allocated to the training set according to 7 : 3. For the sake of enhancing the universality of the results and reducing the chance, in the process of assigning the training set and the validation set, we did not choose to distribute each pulse in equal proportion according to the 10 types of pulses but chose to do all the data randomly assigned. However, due to the small sample size, it may result in less pulse wave data of a certain type in the test set, which may lead to a lower performance in the evaluation parameters of the results, but they are all stable at more than 80%. If you can expand the data capacity or increase the number of training times, this phenomenon can also be greatly improved.

Compared with the evaluation parameters, the confusion matrix can more intuitively see the classification results of each type of pulse wave.

The confusion matrix is an indicator for judging the results of the model and is part of the model evaluation indicators. The confusion matrix is mostly used to indicate the quality of the classifier. The confusion matrix is also called the error matrix [22]. In the error matrix, each row of the matrix stands for the class prediction of the data by the classifier, and each column of the matrix represents the true category to which the version belongs [23]. The reason why it is called the confusion matrix is that it can intuitively see whether the results of the samples are confused in the results of the machine learning output.

**Figure 14:**
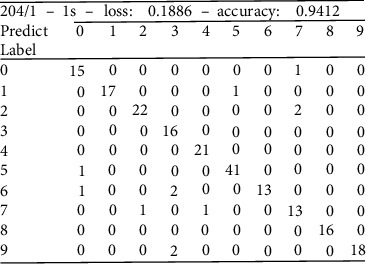
Confusion matrix of the CNN model (test set).

From the diagonal of the confusion matrix in Figure 14, it can intuitively see the number of pulse prediction pairs of each type and the result of erroneous prediction. It can adjust the deep learning model or adjust the feature extraction according to the part of the result set that is wrongly predicted. Evaluation parameters can also be calculated based on the confusion matrix.

## 5. Conclusions

This research collects pulse signals through self-made sensors. Then, the pulse signal is subjected to wavelet transform to denoise. Analyze the pulse signal in time frequencies and perform feature extraction. The time frequencies include the extraction of pulse signal feature points, the energy extraction of pulse wavelet coefficients, and the energy extraction of the HHT transform. Hope to use statistical methods to analyze the wavelet coefficients in each layer in detail, we can study their relationship with specific diseases and promote the objective development of pulse diagnosis. In the signal acquisition, a total of ten different pulse signals are collected, and this data is used to classify the pulse signals based on deep learning. The classification model selects the CNN model. The initial signal is classified by signal segmentation and labeling. Build a one-dimensional CNN model, utilize the model to train the data, and then get a pulse signal classifier with an accuracy of about 95%.

In the future, we consider combining a CNN module and a VT module proposed in [24] for local and global feature extraction, respectively, to improve the model performance. Meanwhile, the strategy used in LCU-net [25] is considered to optimize the model, where the dense CRF is applied to reduce the memory cost. Besides, we can use MRF and CRF methods in [26] to mask the annotations of the various groups of imaging functions and regions of interest with the data augmentation. Furthermore, we can also apply the GasHisTransformer [27] to capture long-range correlation considering the global and local associations of the pulse signal in a unified context.

## Figures and Tables

**Table 1:** Band range of wavelet coefficients of each layer decomposed by 9-level wavelets.

Wavelet coefficients	*cD*_1_	*cD*_2_	*cD*_3_	*cD*_4_	*cD*_5_	*cD*_6_	*cD*_7_	*cD*_8_	*cD*_9_	*cA*_1_
Hz	50-100	25-50	12.5-25	6.3-12.5	3.1-6.3	1.6-3.1	0.8-1.6	0.4-0.8	0.2-0.4	0-0.2

**Table 2:** Normalized eigenvector *T*_0_ of the wavelet energy.

	*E*_*cA*5_	*E*_*cD*5_	*E*_*cD*4_	*E*_*cD*3_	*E*_*cD*2_	*E*_*cD*1_
Sample 1	0.9584	0.0386	0.0028	0.0002	0.0000	0.0000
Sample 2	0.9273	0.0641	0.0084	0.0002	0.0000	0.0000
Sample 3	0.9832	0.0134	0.0034	0.0000	0.0000	0.0000
Sample 4	0.9752	0.0201	0.0046	0.0001	0.0000	0.0000
Sample 5	0.9943	0.0048	0.0008	0.0001	0.0000	0.0000
Sample 6	0.9813	0.0162	0.0024	0.0001	0.0000	0.0000

**Table 3:** The arrangement of training and test datasets.

Dataset/class	Train	Test	Total
Changmai	43	16	59
Chimai	31	18	49
Dongmai	55	24	79
Huamai	47	16	63
Huanmai	42	21	63
Jimai	88	42	130
Pingmai	38	16	54
Shimai	45	15	60
Weimai	34	16	50
Xuanmai	50	20	70
Total	473	204	677

**Table 4:** The evaluation metrics for pulse signal classification.

Metric	Definition	Metric	Definition
Precision	$\text{Precision}=\frac{\text{TP}}{\text{TP}+\text{FP}}$	Recall	Recall=TPTP+FN
	$\text{F}1=\frac{2\times \text{Precision}\times \text{Recall}}{\text{Precision}+\text{Recall}}$		
F1-score			

## Data Availability

The data used to support the findings of this study are available from the first author upon request.
